# Guidelines and Policies as Levers in Managing Carpal Tunnel Release Demand

**DOI:** 10.1177/23814683261477058

**Published:** 2026-08-24

**Authors:** Mikael Hytönen, Aukusti Savolainen, Aleksi Reito, Yrjänä Nietosvaara, Ville Mattila, Matti Juntunen, Mikko P. Räisänen

**Affiliations:** 1 University of Eastern Finland, School of Medicine, Kuopio, Finland; 2 Kuopio University Hospital, Department of Orthopaedics, Traumatology and Hand Surgery, Kuopio, Finland; 3 Tampere University Hospital, Centre for Musculoskeletal Diseases, Tampere, Finland; 4 Kuopio University Hospital, Department of Paediatric Surgery. Kuopio, Finland; 5 Tampere University, Faculty of Medicine and Health Technology, Tampere, Finland

**Keywords:** carpal tunnel, guidelines, hand, register study, surgery

## Abstract

**Background:**

National guidelines and health policy measures can influence both demand for and delivery of elective surgery as well as the setting in which procedures are performed. Carpal tunnel release (CTR), one of the most common elective operations, provides a useful case for examining these effects. This study addresses 2 key research objectives: 1) the evaluation of the impact of Finland’s 2007 guideline requiring preoperative electromyoneurography on CTR incidence trends and 2) the assessment of trends in the adoption of procedural room (PR) surgery in university hospitals.

**Methods:**

We used Finnish National Hospital Discharge Register (NHDR) data to identify all CTR procedures between 1997 and 2021. The years 2020 to 2021 were excluded due to the COVID-19 pandemic. Negative binomial regression models with population offset were used to estimate annual changes in incidence, adjusted for age and sex. Age- and sex-standardized incidence rates were calculated using direct standardization. Pre- and postguideline period analyses were performed. University hospital records (2017–2025) were analyzed to determine the rates of PR-based CTR.

**Results:**

Between 1997 and 2019, 181,552 CTR procedures were performed in Finland. The age- and sex-standardized incidence increased from 91.9 to 233 per 100,000 person-years. From 1997 to 2007, the incidence of CTR increased by 7.5% annually (*P* < 0.001), whereas after the 2007 guideline, the increase slowed to 1.2% annually (*P* < 0.001). From 2017 to 2025, 4 of 5 Finnish university hospitals shifted most CTR operations from operating theaters to PRs, demonstrating rapid institution-level adaptation.

**Conclusion:**

The 2007 national guideline was associated with a marked reduction in CTR incidence growth. The move to PR-based CTR in university hospitals illustrates a high-impact policy strategy for reducing costs, reallocating operating theater capacity, and promoting more sustainable surgical care.

**Highlights**

- For policy makers, this study indicates that implementing a national guideline requiring preoperative electrodiagnostic confirmation before carpal tunnel release (CTR) surgery can effectively moderate procedure growth.
- For hospital administrators, the rapid shift of CTR procedures from traditional operating theater to outpatient procedural room in major hospitals demonstrates a feasible, high-impact strategy to lower costs and improve the use of surgical resources.
- Health policy makers should anticipate continued growth in demand for CTR driven by population aging, particularly in the 50- to 59-y age group, and incorporate this trend into long-term capacity planning.

## Introduction

Carpal tunnel syndrome (CTS) stands as the most common peripheral nerve entrapment syndrome worldwide.^1^ It occurs when the median nerve is compressed at carpal tunnel—a rigid closed structure that originates at the level of the distal wrist crease, extending up to 3 cm distally to the palm,^2^ and contains the median nerve and finger flexor tendons.^1^ The internal pressure of the carpal tunnel has been reported to be elevated in patients with CTS. This is believed to cause nerve ischemia^3^ and to impair the blood–nerve barrier, resulting in a cascade of swelling, inflammation, and scarring.^4^ While rarely attributed to a single cause, risk factors include age and female gender,^5^ hypothyroidism,^6^ pregnancy,^7^ menopause,^8^ rheumatoid arthritis,^9^ osteoarthritis,^10^ obesity,^11^ diabetes,^12^ and occupational factors such as vibrating tools or high-grip-force tasks.^13^ The diagnosis of CTS is a combination of patient history, physical examination, and electrodiagnostic tests.^14^ The American Academy of Orthopaedic Surgeons (AAOS) recommends nerve conduction velocity studies, such as electroneuromyography, prior to operative treatment.^14^

The prevalence of CTS has been reported to be as high as 2.7% in the general population.^15^ In Finland, the incidence of CTS is 105 per 100,000 person-years for men and 197 per 100,000 person-years for women.^5^ In the United States, the incidence has been reported as 258 per 100,000 person-years for men and 428 per 100,000 person-years for women.^16^ More than 60% of diagnosed CTS cases in Finland are treated surgically with carpal tunnel release (CTR), across various levels of hospitals.^5^ Observational studies have indicated that CTR could be beneficial in the treatment of CTS.^17^ In the United States, up to 600,000 patients undergo CTR each year,^18^ and as the average cost of CTR is about US$3,500 per procedure,^19^ this results in yearly costs of more than US$2 billion. In Finland, CTR results in annual costs of €20.7 million.^20^

The incidence of CTS has been on the rise, leading to an increase in the rate of CTR procedures.^16,21–25^ Predictions suggest that this trend will persist in the future.^22^ In the mid-2000s, the Finnish Society for Surgery of the Hand recommended that all patients undergoing CTR should also undergo preoperative electroneuromyography to ensure a proper diagnosis of CTS. The Finnish Current Care Guidelines for “strain-related disorders of the hand and forearm” from 2007 included this recommendation.^26^

When implemented correctly, guidelines can help to manage health care costs^27^ while benefitting the patient, professionals, and entire health care systems.^28^ There have been studies on the effect of guidelines on the incidence of surgery. For example, the trend of total colectomy had been increasing from 2000 to 2006 in the United States, but after the new guideline from American Society of Colon and Rectal Surgeons in 2006, the rate of this increase slowed down and in certain age groups halted.^29^ With thyroid surgery, after the American Thyroid Association released new guidelines, the rate of total thyroidectomy drastically decreased.^30^ However, no studies to date have examined the effect of guidelines on CTR incidence.

Over the years, surgical techniques and CTR safety have improved. As a result, CTR is now more often performed in outpatient procedural rooms under local anesthesia rather than in operating theaters with regional or general anesthesia.^18,31,32^ Earlier studies indicate that both options are equally safe,^33^ but procedural rooms in outpatient clinics can deliver treatment at lower cost.^34^ There has been a clear need for more cost-effective ways to produce health care services since the health care expenditures have increased substantially.^35^ Shifting CTR from operating theaters to procedural rooms could alleviate the strain on society’s limited health care resources.

Health care decision makers face a dual challenge: ensuring timely access to CTR while managing rising procedure volumes, associated costs, and surgical resource constraints. This study addresses 2 key research questions:

1. What has been the effect of diagnostic guideline changes on CTR incidence trends?
2. How has the adoption of procedural room–based CTR evolved in university hospitals?

## Methods

This retrospective register study adheres to the “The Strengthening the Reporting of Observational Studies in Epidemiology (STROBE) Statement”^36^ guidelines (Supplementary Table S1). We analyzed the yearly nationwide incidence of CTR between 1997 and 2021. However, data after 2019 were excluded due to the COVID-19 pandemic. Data were collected from the Finnish National Hospital Discharge Registry (NHDR). Established in 1967, the Finnish NHDR compiles comprehensive data on patients’ demographics, including age and sex, duration of hospital stays, primary and secondary diagnoses, as well as all operations. Reporting data to the NHDR is mandatory for both public and private hospitals, ensuring comprehensive coverage across Finland. The NHDR’s validity is well documented, with studies consistently attesting to its accuracy and thoroughness in capturing rates of surgical procedures.^37–41^ Population data for age groups in the investigated period were sourced from Statistics Finland,^42^ a government-funded population database.

All adult patients with surgical procedure codes for CTR in the Nordic Medico-Statistical Committee Classification of Surgical Procedures^43^ (NCSP, Finnish version), specifically codes ACC51, ACC59, ACC91, or ACC99, accompanied by *International Classification of Diseases* code (ICD-10)^44^ G56.0, were included in this study. In addition, patients with surgical code ACC51 in combination with ICD-10 codes G56.8 or G56.9 were included. The use of ICD-10 began in Finland in 1996, 1 y prior to our study period. Detailed procedure and diagnosis codes are provided in Supplementary Table S2.

In addition, we gathered the numbers of CTR procedures performed in operating theaters and procedural rooms from 2017 to 2025 in all 5 Finnish university hospitals. Data were collected from the clinical information systems of the hospitals. In one location, procedural room–based CTR was piloted from May 2025 onward, and due to changes in hospital infrastructure, normal procedure volumes were not reached until late 2025. Thus, there were no descriptive figures available from this location, and data from 4 locations were reported.

Our outcomes were the annual CTR incidence rate per 100,000 person-years in Finland before and after the 2007 guideline and the percentage of CTR procedures performed in outpatient clinic procedure rooms.

Sex was classified as male or female according to registry records, which use binary coding. Age was analyzed in predefined categories (18–39, 40–49, 50–59, 60–69, 70–79, and 80+ y). These variables were included to assess potential demographic differences in CTR incidence trends.

None of the funding bodies had a role in planning, data collection, analysis, or writing of the article.

### Statistical Analysis

We analyzed the annual incidence of CTR per 100,000 person-years. Incidence was calculated using NHDR data. Continuous data were presented as mean with standard deviation (SD) and range (minimum and maximum). Incidence was also calculated by sex and age groups (18–39, 40–49, 50–59, 60–69, 70–79, and 80+ y).

To account for changes in population age and sex distribution over time, we calculated age- and sex-standardized incidence rates. This was done using the direct standardization method with the mean Finnish population structure during the study period (1997–2019) as the standard population.

Changes in the annual incidences were modeled using negative binomial regression to account for potential overdispersion in count data for the overall study period as well as pre- (1997–2007) and postguideline (2008–2019). In addition, an interaction model was performed between year and the guideline period to assess possible differences in annual trends of incidence rates. Population size was included as a log offset in the models to estimate incidence rate ratios. The models were adjusted for age group and sex. Results are reported with 95% confidence intervals (CIs) and *P* values.

Procedural room adoption rates were calculated as the proportion of CTR performed outside operating theaters annually. All statistical analyses were performed using R Statistical Software (v4.5.5; R Core Team 2025) and appropriate packages.

## Results

### Standardized CTR Incidence

**Figure 1.**
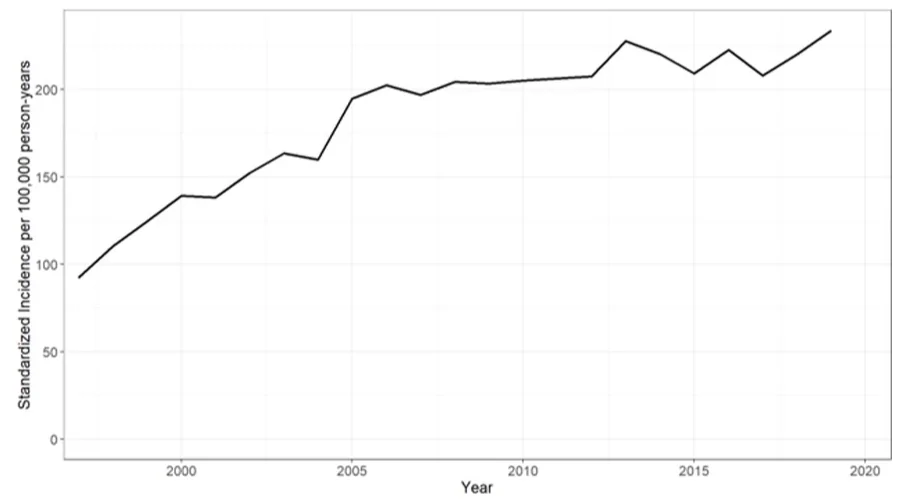
Age- and sex-adjusted carpal tunnel release rates (per 100,000 person-year) by year.

Between 1997 and 2021, a total of 198,113 CTR procedures were performed. Over the years of interest (from 1997 to 2019), 181,552 procedures were performed. The annual CTR numbers are presented in Supplementary Table S3. In total, 58,530 CTRs (32.2%) were performed on males and 123,022 (67.8 %) on females. The age- and sex-standardized incidence increased over the study period from 91.9 to 233 per 100,000 person-years (Figure 1).

### CTR Incidence Trends

**Figure 2.**
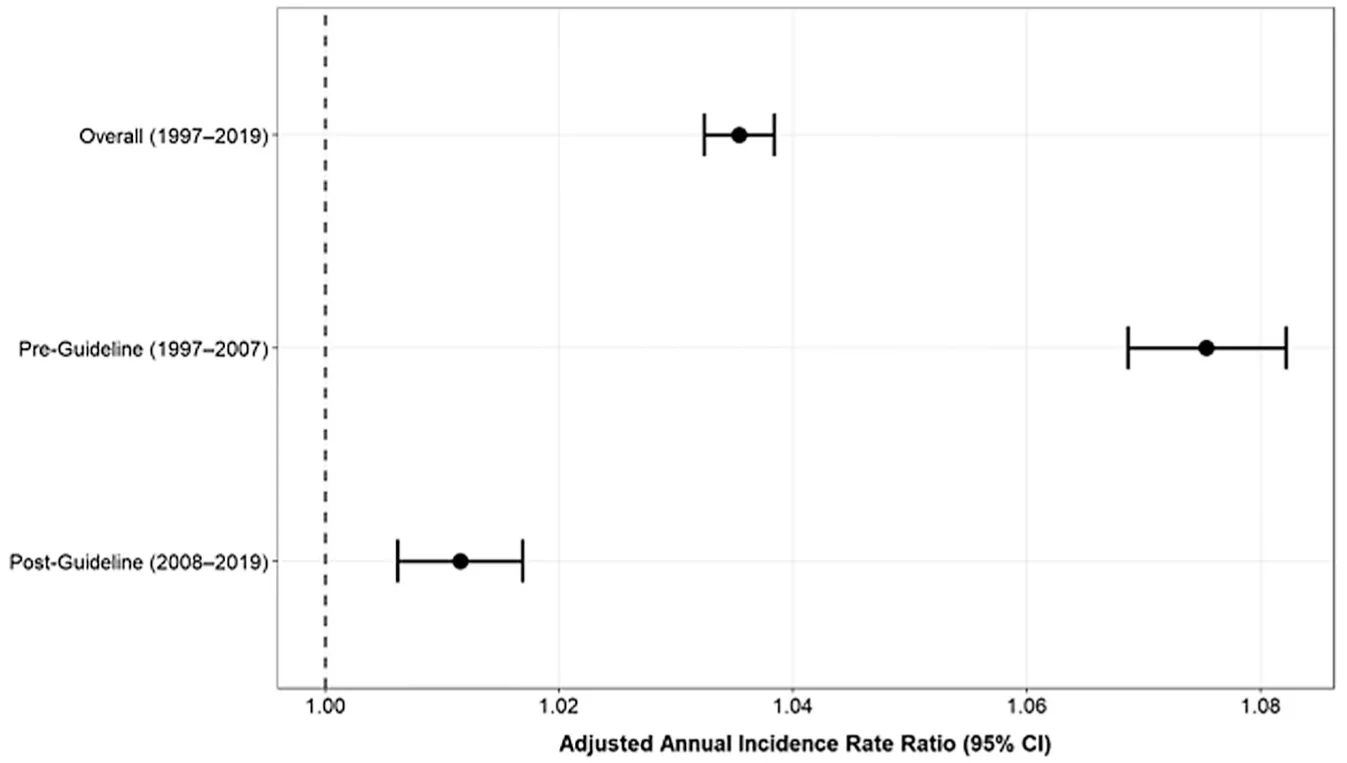
Forest plot of annual carpal tunnel release incidence rate ratios from 1997 to 2019 and before (1997–2007) and after (2008–2019) the guideline.

Using age- and sex-adjusted negative binomial regression analysis, the CTR incidence increased by 3.5% annually. The preguideline increase in CTR incidence from 1997 to 2007 was 7.5% per year. After the guideline from 2008 onward, the rate decreased to only 1.2% per year (Figure 2). An interaction analysis showed that the guideline implementation resulted in a drastic decrease in annual CTR incidence (interaction incidence rate ratio = 1.062). CIs and *P* values are shown in Table 1.

**Table 1.** Negative Binomial Regression Analysis of the Incidence of Carpal Tunnel Release (with Population Offset) with Pre- and Postguideline Analysis.

Analysis	IRR	95% CI	*P* Value
Overall 1997–2019	1.035	1.032–1.038	<0.001
Pre (1997–2007)	1.079	1.069–1.082	<0.001
Post (2008–2019))	1.012	1.0062–1.017	<0.001
Pre vs post interaction	1.062	1.053–1.072	<0.001

CI, confidence interval; IRR, incidence rate ratio.

### CTR Incidence by Sex and Age Group

**Figure 3.**
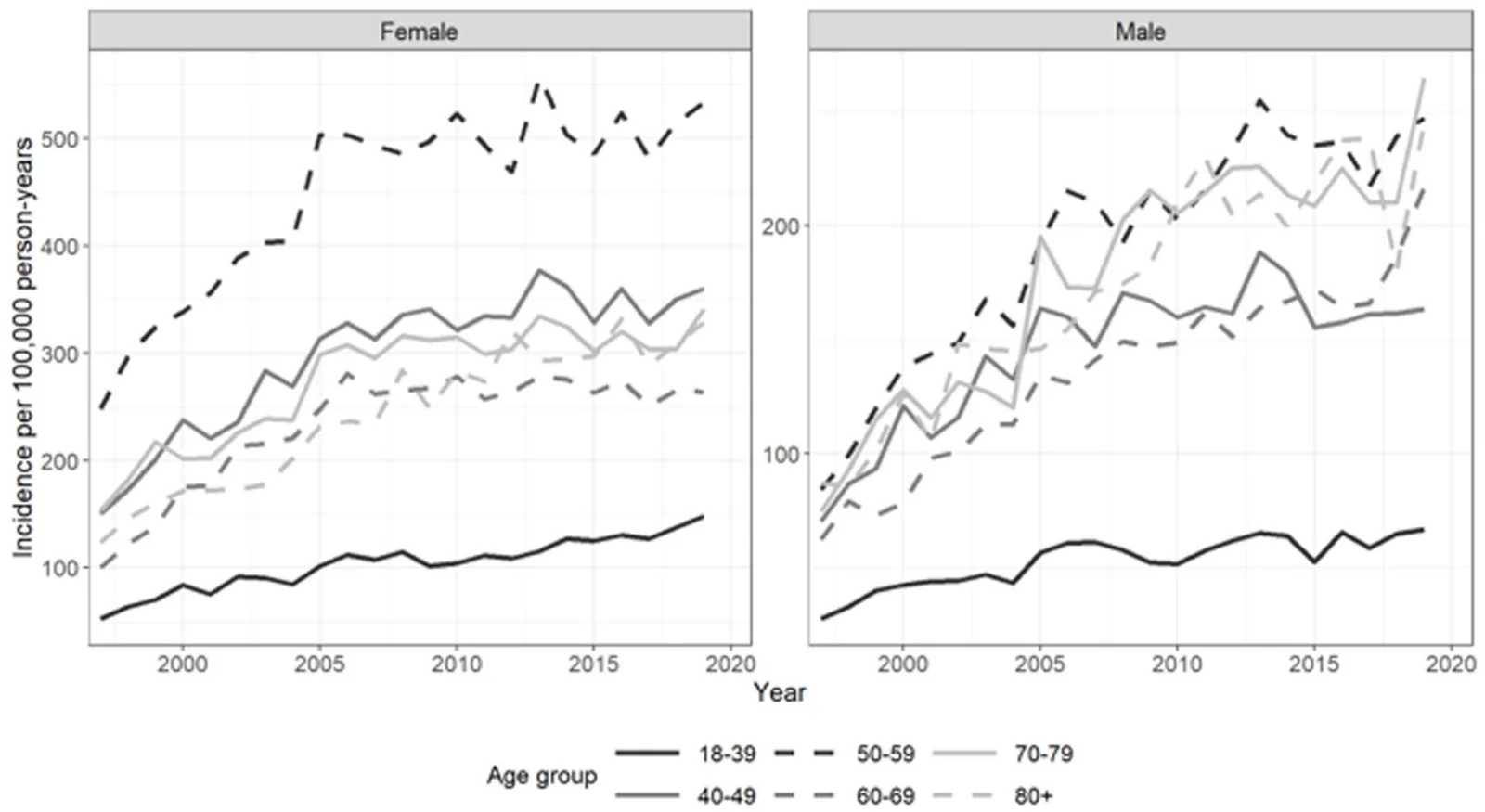
Carpal tunnel release incidence from 1997 to 2019 by sex and age group.

The analysis of CTR incidence by sex and age group revealed similar results, with increasing trends over the years. The highest incidence was in the 50- to 59-y age group (SD = 147, range = 84.1 to 557), and the lowest incidence was in the 18- to 39-y age group (SD = 78.3, range = 27.6 to 148). Development of CTR incidence by sex and age group described in Figure 3.

### CTR in Procedural Rooms

**Figure 4.**
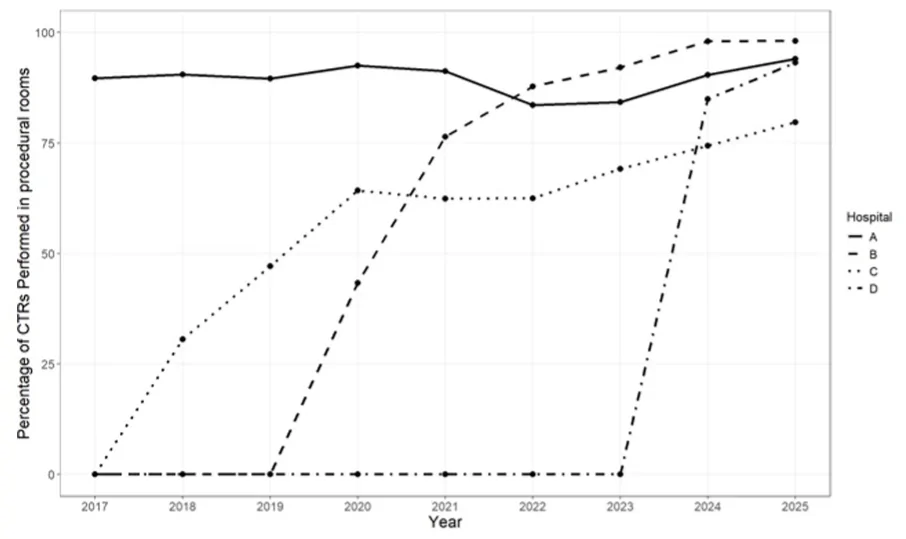
Proportion of carpal tunnel releases performed in procedural rooms compared with total procedures in 4 Finnish university hospitals, 2017 to 2025.

We analyzed the trend of CTR in procedural rooms from 2017 to 2025. In the beginning of the study period, CTR was performed predominantly in procedural rooms already in 1 university hospital. Three additional hospitals started shifting CTR out of operating theaters during the investigated time period (Figure 4).

## Discussion

In a previous study, it was determined that the lifetime prevalence of CTR in Finland was 3.1%, with an incidence rate of 173 per 100,000 person-years from 2001 to 2011.^45^ Our results extend this knowledge by providing temporal depth: the incidence increased from 91.9 to 233 per 100,000 person-years from 1997 to 2019. A clear increasing trend over time was discovered. However, the introduction of Finland’s diagnostic guideline in 2007 had a significant impact on CTR incidence: the annual increase slowed from 7.5% before 2007 to 1.2% afterward, and this change in the trend was statistically significant. Standardization demonstrated similar trends across sex and age groups over time, confirming the robustness of this effect. We also observed a rapid and marked shift in CTR setting inside large university hospitals. This suggests that Finland’s health care system has adapted to deliver CTR more efficiently in both clinical and economic terms.

Our results suggest that the guideline effectively slowed the rate at which the CTR incidence was increasing. This aligns with evidence from other surgical domains, where structured guidelines have influenced operative rates.^29,30^ For health care decision makers, these results highlight the role of well-designed diagnostic policies to manage surgical demand while safeguarding patient outcomes. Nevertheless, the CTR incidence trend remains upward, and it is likely driven by multiple factors, including greater public awareness,^16^ improved health care access, an aging population, and rising rates of obesity—a known risk factor for CTS.^11,46^ The peak incidence occurs in the 50- to 59-y age group, which is projected to grow substantially in Finland by 2040,^47^ suggesting that demand for CTR will continue to increase. This mandates further allocation of health care resources and highlights the need for careful planning in their use by health care decision makers.

With rising health care costs,^35,48^ cost-effective solutions are urgently needed. One such approach is shifting procedures from operating theaters to outpatient clinics.^49–52^ Many surgeons oppose this change, citing concerns about higher infection rates, although evidence does not support this claim.^53^ In Canada, Peters and Giuffre^54^ reported that up to 88% of surgeons perform CTR in procedural rooms. In contrast, Kammien et al^55^ found that only 4% of CTR procedures in the United States were performed in procedural rooms in 2020, although this was an increase from 1% in 2010. It appears that in Finland, even large institutions, such as university hospitals, are capable of quickly adopting cost-effective solutions. This finding encourages policy makers to recommend outpatient CTR. However, we did not investigate the possible reasons for the differences in procedural room CTR adoption, but as far as we know, the adoption was delayed mainly due to hospital setting limitations (eg, no available room for a new procedural room).

Globally, studies indicate an increasing CTR incidence. In Sweden, Tadjerbashi et al^21^ observed a 5% to 6% annual rise from 2001 to 2009, while Gelfman et al^16^ reported a 2% annual increase in Minnesota, United States, from 1981 to 2005. In France, Oberlin^24^ found a 6% annual increase (1993–1999), followed by a 25% rise in CTR operations from 1999 to 2008 (Tuppin et al^23^). In the United Kingdom, Burton et al^25^ noted an initial increase in CTR rates (1993–2013), although recent trends show a decline in surgeries per patient. Bebbington and Furniss^22^ reported a 3% annual increase in England (2000–2011) and predicted a 99% rise by 2030. Many of these results closely resembled the situation of Finland prior to the 2007 guideline. However, after the guideline, the growth rate slowed by more than 6-fold.

Managing global warming across all sectors of society is crucial, and health care is no exception. Up to 10% of a nation’s greenhouse gas emissions have been attributed to the health care sector.^56^ Surgery, particularly through operating theater maintenance, generates a significant portion of a hospital’s total waste.^57^ Performing operations in a procedural room instead of an operating theater reduces waste per procedure,^58,59^ making it a recommended approach for environmentally sustainable surgery^57^ and a preferable option for decision makers to recommend.

One of the key strengths of this study is the nationwide utilization of NCSP operation codes (ACC51, ACC59, ACC91, and ACC99) in conjunction with CTR. Consequently, we can confidently assert that we have captured nearly all CTR procedures performed in operating theaters, as documented in the Finnish NHDR, a consistently validated and reliable source.^38,39,41^ Furthermore, Finland’s universal health care system ensures medical care is accessible to all individuals, allowing for comprehensive coverage of the entire adult population in this study.

Our data represent the total number of CTR procedures performed in Finland. However, a limitation of this study is that we cannot accurately report the number of recurrent cases operated on. That said, CTS recurrence is rare^60^ and unlikely to introduce significant bias. Another limitation is that NHDR data include only procedures performed in operating theaters, preventing a thorough comparison across subregions, such as hospital districts, where some CTR procedures take place in outpatient clinic procedural rooms. However, as our limited sample of university hospitals indicates, the adoption of procedure rooms happened, for the most part, in the late 2010s and therefore should not pose a significant threat to our analysis of overall nationwide incidence. A limitation from the registry origin of the data is that in addition to sex and age group, other individual-level variables such as socioeconomic status were not available. In addition, due to limitations in electronic health record systems, it was not possible to collect data on CTR procedures performed in procedural rooms across all Finnish hospitals.

Future research should focus on analyzing the factors contributing to the rising incidence of CTR and evaluating their validity. In addition, other treatment methods, for example, more detailed review of conservative treatment method possibilities, and protocols of treatment for CTS should be evaluated. A critical examination of these factors could support more informed decisions regarding the allocation of health care resources in the future.

## Conclusion

The incidence of CTR increased from 1997 to 2019. The increase from 1997 to 2007 was similar to those in many other Western countries. After the 2007 guideline, the rate of increase remained significantly lower than in other Western countries. The number of CTR procedures performed in procedural rooms increased in university hospitals during the study period, freeing time and resources in the operating theaters. For decision makers, this study highlights 2 important strategies for managing surgical demand: robust diagnostic verification to ensure appropriate patient selection and the adoption of cost-effective, environmentally responsible surgical settings.

## Supplemental Material

sj-docx-1-mpp-10.1177_23814683261477058 – Supplemental material for Guidelines and Policies as Levers in Managing Carpal Tunnel Release Demand Supplemental material, sj-docx-1-mpp-10.1177_23814683261477058 for Guidelines and Policies as Levers in Managing Carpal Tunnel Release Demand by Mikael Hytönen, Aukusti Savolainen, Aleksi Reito, Yrjänä Nietosvaara, Ville Mattila, Matti Juntunen and Mikko P. Räisänen in MDM Policy & Practice

## References

[1] Padua L , et al. Carpal tunnel syndrome: clinical features, diagnosis, and management. Lancet Neurol. 2016;15:1273–1284. 27751557 10.1016/S1474-4422(16)30231-9

[2] Standring S. Gray’s Anatomy: the anatomical basis of clinical practice. 42nd ed. Elsevier; 2021.

[3] Aboonq MS. Pathophysiology of carpal tunnel syndrome. Neurosciences (Riyadh). 2015;20:4–9. 25630774 PMC4727604

[4] Mackinnon SE. Pathophysiology of nerve compression. Hand Clin. 2002;18:231–241. 12371026 10.1016/s0749-0712(01)00012-9

[5] Hulkkonen S , et al. Incidence and operations of median, ulnar and radial entrapment neuropathies in Finland: a nationwide register study. J Hand Surg Eur Vol. 2020;45:226–230. 10.1177/1753193419886741 31739732

[6] Shiri R. Hypothyroidism and carpal tunnel syndrome: a meta-analysis. Muscle Nerve. 2014;50:879–883. 25204641 10.1002/mus.24453

[7] Padua L , et al. Systematic review of pregnancy-related carpal tunnel syndrome. Muscle Nerve. 2010;42:697–702. 20976778 10.1002/mus.21910

[8] Kaplan Y Kurt SG Karaer H. Carpal tunnel syndrome in postmenopausal women. J Neurol Sci. 2008;270:77–81. 18325536 10.1016/j.jns.2008.02.003

[9] George RJ , et al. Carpal tunnel syndrome as an early underrecognized feature of rheumatoid arthritis: a population-based study of carpal tunnel syndrome occurrence before and after rheumatoid arthritis incidence. Arthritis Care Res. 2025;77(11):1332–1339. 10.1002/acr.25566 PMC12353601 40322852

[10] Chen S , et al. Risk of carpal tunnel syndrome among patients with osteoarthritis: a US population-based study. BMC Musculoskelet Disord. 2024;25:468. 38879540 10.1186/s12891-024-07459-1 PMC11179394

[11] Shiri R Pourmemari MH Falah-Hassani K Viikari-Juntura E. The effect of excess body mass on the risk of carpal tunnel syndrome: a meta-analysis of 58 studies. Obes Rev. 2015;16:1094–1104. 26395787 10.1111/obr.12324

[12] Pourmemari MH Shiri R. Diabetes as a risk factor for carpal tunnel syndrome: a systematic review and meta-analysis. Diabet Med. 2016;33:10–16. 26173490 10.1111/dme.12855

[13] Shiri R Miranda H Heliövaara M Viikari-Juntura E. Physical work load factors and carpal tunnel syndrome: a population-based study. Occup Environ Med. 2009;66:368–373. 19451144 10.1136/oem.2008.039719

[14] Keith MW , et al. Diagnosis of carpal tunnel syndrome. J Am Acad Orthop Surg. 2009;17:389–396. 19474448 10.5435/00124635-200906000-00007 PMC5175465

[15] Atroshi I , et al. Prevalence of carpal tunnel syndrome in a general population. JAMA. 1999;282:153–158. 10411196 10.1001/jama.282.2.153

[16] Gelfman R , et al. Long-term trends in carpal tunnel syndrome. Neurology. 2009;72:33–41. 19122028 10.1212/01.wnl.0000338533.88960.b9 PMC2633642

[17] Klokkari D Mamais I. Effectiveness of surgical versus conservative treatment for carpal tunnel syndrome: a systematic review, meta-analysis and qualitative analysis. Hong Kong Physiother J. 2018;38:91–114. 30930582 10.1142/S1013702518500087 PMC6405353

[18] Fajardo M Kim SH Szabo RM. Incidence of carpal tunnel release: trends and implications within the United States ambulatory care setting. J Hand Surg Am. 2012;37:1599–1605. 22727925 10.1016/j.jhsa.2012.04.035

[19] Gabrielli AS Lesiak AC Fowler JR. The direct and indirect costs to society of carpal tunnel release. Hand (N Y). 2020;15:NP1–NP5. 10.1177/1558944718810855 PMC7076612 30417688

[20] Lampainen K , et al. Registry cost description of carpal tunnel release in Finland in 2011–2015. BMJ Open. 2024;14:e080855. 10.1136/bmjopen-2023-080855 PMC11227770 38960470

[21] Tadjerbashi K Åkesson A Atroshi I. Incidence of referred carpal tunnel syndrome and carpal tunnel release surgery in the general population: increase over time and regional variations. J Orthop Surg (Hong Kong). 2019;27:2309499019825572. 10.1177/2309499019825572 30798784

[22] Bebbington E Furniss D. Linear regression analysis of Hospital Episode Statistics predicts a large increase in demand for elective hand surgery in England. J Plast Reconstr Aesthet Surg. 2015;68:243–251. 25455287 10.1016/j.bjps.2014.10.011 PMC4315884

[23] Tuppin P Blotière P-O Weill A Ricordeau P Allemand H. Syndrome du canal carpien opéré en France en 2008: caractéristiques des malades et de leur prise en charge. Rev Neurol. 2011;167:905–915. 22035728 10.1016/j.neurol.2011.05.010

[24] Oberlin M . Centre hospitalier de Villeneuve Saint Georges et ENSP-Groupe IMAGE - Marie-Claude MOUQUET (DREES) (2002). Les interventions de chirurgie fonctionnelle : une activité programmée importante mais hétérogène. Études et Résultats, 194.

[25] Burton CL Chen Y Chesterton LS van der Windt DA. Trends in the prevalence, incidence and surgical management of carpal tunnel syndrome between 1993 and 2013: an observational analysis of UK primary care records. BMJ Open. 2018;8:e020166. 10.1136/bmjopen-2017-020166 PMC6020969 29921681

[26] Käden ja kyynärvarren rasitussairaudet. Käypä hoito -suositus. Suomalaisen Lääkäriseuran Duodecimin ja Suomen Työterveyslääkäriyhdistyksen asettama työryhmä. [accessed 2024 Aug 20]. https://www.duodecimlehti.fi/duo96610

[27] Shapiro DW Lasker RD Bindman AB Lee PR. Containing costs while improving quality of care: the role of profiling and practice guidelines. Annu Rev Public Health. 1993;14:219–241. 8323588 10.1146/annurev.pu.14.050193.001251

[28] Woolf SH Grol R Hutchinson A Eccles M Grimshaw J. Potential benefits, limitations, and harms of clinical guidelines. BMJ. 1999;318:527–530. 10024268 10.1136/bmj.318.7182.527 PMC1114973

[29] Strassle PD , et al. Rates of elective colectomy for diverticulitis continued to increase after 2006 guideline change. Gastroenterology. 2019;157:1679–1681.e11. 10.1053/j.gastro.2019.08.045 PMC6878190 31499038

[30] Toumi A , et al. Trends in thyroid surgery and guideline-concordant care in the United States, 2007–2018. Thyroid. 2021;31:941–949. 33280499 10.1089/thy.2020.0643 PMC8215427

[31] Munns JJ Awan HM. Trends in carpal tunnel surgery: an online survey of members of the American society for surgery of the hand. J Hand Surg. 2015;40:767–771.e2. 10.1016/j.jhsa.2014.12.046 25747738

[32] Rodríguez-Martínez P , et al. Carpal tunnel release surgery: small-area variation and impact of ambulatory surgery in the autonomous region of Valencia, Spain. Gaceta Sanitaria. 2013;27:214–219. 23218975 10.1016/j.gaceta.2012.07.008

[33] LeBlanc MR , et al. Is main operating room sterility really necessary in carpal tunnel surgery? A multicenter prospective study of minor procedure room field sterility surgery. Hand (N Y). 2011;6:60–63. 22379440 10.1007/s11552-010-9301-9 PMC3041895

[34] Alter T Warrender W Liss F Ilyas AM. A cost analysis of carpal tunnel release surgery performed wide awake versus under sedation. Plast Reconstr Surg. 2018;142:1532–1538. 30188472 10.1097/PRS.0000000000004983

[35] Harris E. Health care costs will comprise about 20% of US economy by 2031. JAMA. 2023;330:210. 10.1001/jama.2023.11953 37379028

[36] von Elm E , et al. The Strengthening the Reporting of Observational Studies in Epidemiology (STROBE) statement: guidelines for reporting observational studies. Lancet. 2007;370:1453–1457. 18064739 10.1016/S0140-6736(07)61602-X

[37] Mattila VM , et al. Declining incidence of surgery for Achilles tendon rupture follows publication of major RCTs: evidence-influenced change evident using the Finnish registry study. Br J Sports Med. 2015;49:1084–1086. 24128757 10.1136/bjsports-2013-092756

[38] Mattila VM , et al. Coverage and accuracy of diagnosis of cruciate ligament injury in the Finnish National Hospital Discharge Register. Injury. 2008;39:1373–1376. 18703187 10.1016/j.injury.2008.05.007

[39] Huttunen TT Kannus P Pihlajamäki H Mattila VM. Pertrochanteric fracture of the femur in the Finnish National Hospital Discharge Register: validity of procedural coding, external cause for injury and diagnosis. BMC Musculoskelet Disord. 2014;15:98. 24655318 10.1186/1471-2474-15-98 PMC4026595

[40] Heinänen M Brinck T Handolin L Mattila VM Söderlund T. Accuracy and coverage of diagnosis and procedural coding of severely injured patients in the Finnish Hospital Discharge Register: comparison to patient files and the Helsinki Trauma Registry. Scand J Surg. 2017;106:269–277. 28537212 10.1177/1457496916685236

[41] Sund R. Quality of the Finnish Hospital Discharge Register: a systematic review. Scand J Public Health. 2012;40:505–515. 22899561 10.1177/1403494812456637

[42] Suomen Virallinen Tilasto (SVT). Väestön tieto- ja viestintätekniikan käyttö [verkkojulkaisu]. Tilastokeskus [Viitattu: 13.5.2024]. Saantitapa: https://stat.fi/tilasto/sutivi

[43] Nordic Medico-Statistical Committee. NOMESCO Classification of Surgical Procedures (NCSP), version 1.15, [Internet]. 2010. Available from: https://urn.kb.se/resolve?urn=urn:nbn:se:norden:org:diva-4620

[44] Terveyden ja hyvinvoinnin laitos (THL). THL - Tautiluokitus ICD-10. https://urn.fi/URN:NBN:fi-fe201205085423

[45] Pourmemari M-H Heliövaara M Viikari-Juntura E Shiri R. Carpal tunnel release: lifetime prevalence, annual incidence, and risk factors. Muscle Nerve. 2018;58:497–502. 29665085 10.1002/mus.26145

[46] Jääskeläinen T Koponen P Lundqvist A Koskinen S. Lifestyle factors and obesity in young adults—changes in the 2000s in Finland. Scand J Public Health. 2022;50:1214–1220. 35130774 10.1177/14034948221075427 PMC9720455

[47] Tilastokeskus. Suomen virallinen tilasto (SVT): Väestön tieto- ja viestintätekniikan käyttö [verkkojulkaisu]. Tilastokeskus. 2024. Saantitapa: https://stat.fi/tilasto/sutivi

[48] Eurostat. Government expenditure on health; [accessed 2024 Oct 28]. https://ec.europa.eu/eurostat/statistics-explained/index.php?title=Government_expenditure_on_health

[49] Lalonde D Martin A. Tumescent local anesthesia for hand surgery: improved results, cost effectiveness, and wide-awake patient satisfaction. Arch Plast Surg. 2014;41:312. 25075350 10.5999/aps.2014.41.4.312 PMC4113687

[50] Kazmers NH Presson AP Xu Y Howenstein A Tyser AR. Cost implications of varying the surgical technique, surgical setting, and anesthesia type for carpal tunnel release surgery. J Hand Surg. 2018;43:971–977.e1. 10.1016/j.jhsa.2018.03.051 PMC6218304 29784549

[51] Starr BW Davenport RO Granzow D Johnson SP Lien JR. Optimizing the use of operating rooms by transitioning common hand surgeries into the office setting. J Hand Surg. 2023;48:217–225. 10.1016/j.jhsa.2022.11.010 36658050

[52] Lin Y-C Chen W-C Chen C-Y Kuo SM. Plate osteosynthesis of single metacarpal fracture: WALANT technique is a cost-effective approach to reduce postoperative pain and discomfort in contrast to general anesthesia and wrist block. BMC Surg. 2021;21:358. 34627230 10.1186/s12893-021-01362-5 PMC8501709

[53] Zhuang T Fox P Curtin C Shah KN. Is hand surgery in the procedure room setting associated with increased surgical site infection? A cohort study of 2,717 patients in the veterans affairs population. J Hand Surg. 2023;48:559–565. 10.1016/j.jhsa.2023.03.001 36973100

[54] Peters B Giuffre JL. Canadian trends in carpal tunnel surgery. J Hand Surg. 2018;43:1035.e1–1035.e8. 10.1016/j.jhsa.2018.02.014 29559326

[55] Kammien AJ , et al. Wide-awake carpal tunnel release in the United States: trends in volume and reimbursement by operative setting. Plast Reconstr Surg. 2024;154(1):143–149. 37535704 10.1097/PRS.0000000000010961

[56] Eckelman MJ Sherman J. Environmental impacts of the U.S. health care system and effects on public health. PLoS One. 2016;11:e0157014. 10.1371/journal.pone.0157014 PMC4900601 27280706

[57] Bravo D Gaston RG Melamed E. Environmentally responsible hand surgery: past, present, and future. J Hand Surg. 2020;45:444–448. 10.1016/j.jhsa.2019.10.031 31928797

[58] Van Demark RE Smith VJS Fiegen A. Lean and green hand surgery. J Hand Surg. 2018;43:179–181. 10.1016/j.jhsa.2017.11.007 29421068

[59] Thiel CL , et al. Minimal custom pack design and wide-awake hand surgery: reducing waste and spending in the orthopedic operating room. Hand (New York, N,Y). 2019;14:271–276. 29183168 10.1177/1558944717743595 PMC6436127

[60] Westenberg RF , et al. Revision carpal tunnel release: risk factors and rate of secondary surgery. Plastic Reconstr Surg. 2020;145:1204. 10.1097/PRS.0000000000006742 32332540

